# Bis(μ-3-nitro­phthalato-κ^2^ *O* ^1^:*O* ^2^)bis­[aqua­(2,2′-bipyridine-κ^2^ *N*,*N*′)copper(II)] dihydrate

**DOI:** 10.1107/S1600536810043229

**Published:** 2010-10-30

**Authors:** Yin-Feng Han, Chen Cheng, Seik Weng Ng

**Affiliations:** a Department of Chemistry and Chemical Engineering, Baoji University of Arts and Science, Baoji 721013, People’s Republic of China; b Department of Biotechnology, Wulanchabu Vocational College, Wulanchabu 012000, Inner Mongolia, People’s Republic of China; c Department of Chemistry, University of Malaya, 50603 Kuala Lumpur, Malaysia

## Abstract

Two 3-nitro­phthalate dianions bridge two water-coordinated 2,2′-bipyridine-chelated Cu^II^ atoms about a center of inversion to generate the title dinuclear compound, [Cu_2_(C_8_H_3_NO_6_)_2_(C_10_H_8_N_2_)_2_(H_2_O)_2_]·2H_2_O. The geometry of the Cu^II^ atom is a distorted square pyramid. Adjacent mol­ecules are linked through the coordinated and solvent water mol­ecules to form a linear ribbon running along the *a* axis of the monoclinic unit cell.

## Related literature

For the isostructural zinc analog, see: Song *et al.* (2007 ▶).
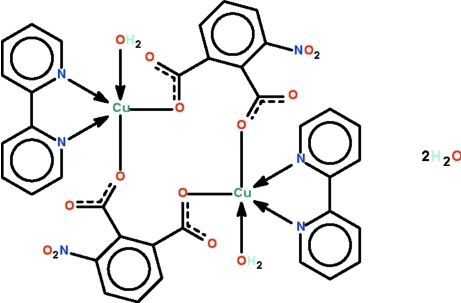


## Experimental

### 

#### Crystal data

[Cu_2_(C_8_H_3_NO_6_)_2_(C_10_H_8_N_2_)_2_(H_2_O)_2_]·2H_2_O
*M* *_r_* = 929.74
Triclinic, 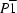

*a* = 7.534 (2) Å
*b* = 10.467 (3) Å
*c* = 12.044 (3) Å
α = 87.835 (2)°
β = 74.911 (3)°
γ = 77.437 (3)°
*V* = 894.9 (4) Å^3^
*Z* = 1
Mo *K*α radiation
μ = 1.28 mm^−1^
*T* = 295 K
0.45 × 0.45 × 0.40 mm

#### Data collection

Bruker SMART APEX diffractometer
Absorption correction: multi-scan (*SADABS*; Sheldrick, 1996 ▶) *T*_min_ = 0.591, *T*_max_ = 0.629
4675 measured reflections
3092 independent reflections
2732 reflections with *I* > 2σ(*I*)
*R*_int_ = 0.023

#### Refinement

*R*[*F* ^2^ > 2σ(*F* ^2^)] = 0.033
*wR*(*F* ^2^) = 0.088
*S* = 1.01
3092 reflections
284 parameters
6 restraints
H atoms treated by a mixture of independent and constrained refinement
Δρ_max_ = 0.54 e Å^−3^
Δρ_min_ = −0.55 e Å^−3^

### 

Data collection: *SMART* (Bruker, 2003 ▶); cell refinement: *SAINT* (Bruker, 2003 ▶); data reduction: *SAINT*; program(s) used to solve structure: *SHELXS97* (Sheldrick, 2008 ▶); program(s) used to refine structure: *SHELXL97* (Sheldrick, 2008 ▶); molecular graphics: *X-SEED* (Barbour, 2001 ▶); software used to prepare material for publication: *publCIF* (Westrip, 2010 ▶).

## Supplementary Material

Crystal structure: contains datablocks global, I. DOI: 10.1107/S1600536810043229/xu5061sup1.cif

Structure factors: contains datablocks I. DOI: 10.1107/S1600536810043229/xu5061Isup2.hkl

Additional supplementary materials:  crystallographic information; 3D view; checkCIF report

## Figures and Tables

**Table 1:** Selected bond lengths (Å)

Cu1—O1	1.967 (2)
Cu1—O3^i^	2.172 (2)
Cu1—O1*w*	1.994 (2)
Cu1—N2	2.029 (2)
Cu1—N3	2.013 (2)

Symmetry code: (i) 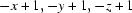.

**Table 2:** Hydrogen-bond geometry (Å, °)

*D*—H⋯*A*	*D*—H	H⋯*A*	*D*⋯*A*	*D*—H⋯*A*
O1*w*—H11⋯O2*w*	0.83 (3)	1.85 (1)	2.660 (3)	166 (3)
O1*w*—H12⋯O4^ii^	0.83 (3)	1.90 (1)	2.718 (3)	168 (3)
O2*w*—H21⋯O4	0.84 (3)	2.04 (2)	2.830 (3)	158 (3)
O2*w*—H22⋯O1^i^	0.84 (3)	2.25 (2)	2.985 (3)	146 (3)
O2*w*—H22⋯O3^i^	0.84 (3)	2.35 (3)	2.977 (3)	132 (3)

Symmetry codes: (i) 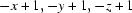; (ii) 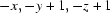.

**Fig. 1.**
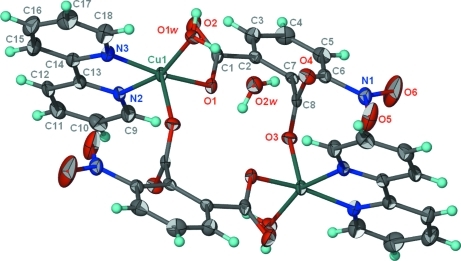
Thermal ellipsoid plot (Barbour, 2001) of dinuclear Cu2(H2O)2(C10H8N2)2(C8H3NO6)2.2H2O at the 50% probability evel; hydrogen atoms are shown as spheres of arbitrary radius.

**Fig. 2.**
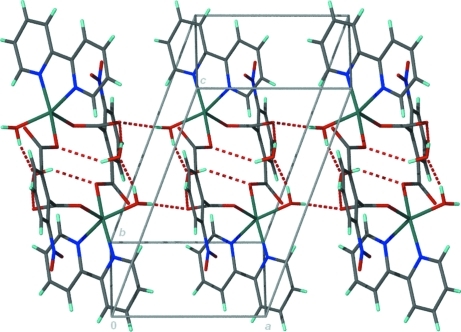
Hydrogen-bonded chain.

## supplementary crystallographic information

### Comment

Dinuclear Zn_2_(H_2_O)_2_(C_10_H_8_N_2_)_2_(C_8_H_3_NO_6_)_2_^.^2H_2_O is reported to exhibit intense fluorescence. In its crystal structure, the 3-nitrophthalate dianion bridges two water-coordinated, 2,2'-bipyridine-chelated zinc atoms about a center-of-inversion; the geometry of the zinc atom is a square pyramid (Song *et al.*, 2007). The present copper analog (Scheme I, Fig. 1) is isostructural, the two compounds crystallizing with matching cell dimensions. Adjacent molecules are connected to the lattice water molecule by hydrogen bonds to form a linear ribbon running along the *a*-axis of the monoclinic unit cell (Fig. 2).

### Experimental

3-Nitrophthalic acid (0.105 g), 2,2'-bipyridine (0.078 g), copper chloride dihydrate (0.085 g) and water (2 ml) were heated at 393 K in a 25 ml, Teflon-lined, stainless-steel Parr bomb for 3 days. Blue crystal were isolated. CH&N elemental analysis. Found: C 46.32, H 3.17, N 9.11%. Calc.: C 46.51, H 3.25, N 9.04%.

### Refinement

Carbon-bound H-atoms were placed in calculated positions (C–H 0.93 Å) and were included in the refinement in the riding model approximation, with *U*(H) set to 1.2*U*(C).

The water H-atoms were located in a difference Fourier map, and were refined with distance restraints of O–H 0.84±0.01 Å and H···H 1.37±0.01 Å; their temperature factors were refined.

### Crystal data

**Table:**

[Cu_2_(C_8_H_3_NO_6_)_2_(C_10_H_8_N_2_)_2_(H_2_O)_2_]·2H_2_O	*Z* = 1
*M**_r_* = 929.74	*F*(000) = 474
Triclinic, *P*1	*D*_x_ = 1.725 Mg m^−^^3^
Hall symbol: -P 1	Mo *K*α radiation, λ = 0.71073 Å
*a* = 7.534 (2) Å	Cell parameters from 3039 reflections
*b* = 10.467 (3) Å	θ = 2.6–28.1°
*c* = 12.044 (3) Å	µ = 1.28 mm^−^^1^
α = 87.835 (2)°	*T* = 295 K
β = 74.911 (3)°	Block, blue
γ = 77.437 (3)°	0.45 × 0.45 × 0.40 mm
*V* = 894.9 (4) Å^3^	

### Data collection

**Table:**

Bruker SMART APEX diffractometer	3092 independent reflections
Radiation source: fine-focus sealed tube	2732 reflections with *I* > 2σ(*I*)
graphite	*R*_int_ = 0.023
φ and ω scans	θ_max_ = 25.0°, θ_min_ = 1.8°
Absorption correction: multi-scan (*SADABS*; Sheldrick, 1996)	*h* = −6→8
*T*_min_ = 0.591, *T*_max_ = 0.629	*k* = −11→12
4675 measured reflections	*l* = −14→14

### Refinement

**Table:**

Refinement on *F*^2^	Secondary atom site location: difference Fourier map
Least-squares matrix: full	Hydrogen site location: inferred from neighbouring sites
*R*[*F*^2^ > 2σ(*F*^2^)] = 0.033	H atoms treated by a mixture of independent and constrained refinement
*wR*(*F*^2^) = 0.088	*w* = 1/[σ^2^(*F*_o_^2^) + (0.0456*P*)^2^ + 0.6587*P*] where *P* = (*F*_o_^2^ + 2*F*_c_^2^)/3
*S* = 1.01	(Δ/σ)_max_ = 0.001
3092 reflections	Δρ_max_ = 0.54 e Å^−^^3^
284 parameters	Δρ_min_ = −0.55 e Å^−^^3^
6 restraints	Extinction correction: *SHELXL97* (Sheldrick, 2008), Fc^*^=kFc[1+0.001xFc^2^λ^3^/sin(2θ)]^-1/4^
Primary atom site location: structure-invariant direct methods	Extinction coefficient: 0.050 (3)

### Fractional atomic coordinates and isotropic or equivalent isotropic displacement parameters (Å^2^)

**Table:**

	*x*	*y*	*z*	*U*_iso_*/*U*_eq_	
Cu1	0.21328 (4)	0.56513 (3)	0.71332 (2)	0.02412 (15)	
N1	0.4799 (3)	0.0616 (2)	0.2658 (2)	0.0343 (6)	
N2	0.2998 (3)	0.4783 (2)	0.84926 (18)	0.0257 (5)	
N3	0.0759 (3)	0.7029 (2)	0.83612 (19)	0.0281 (5)	
O1	0.3115 (3)	0.41035 (18)	0.61015 (15)	0.0280 (4)	
O2	0.0466 (3)	0.3577 (2)	0.71082 (17)	0.0409 (5)	
O3	0.5866 (2)	0.32313 (18)	0.38170 (15)	0.0288 (4)	
O4	0.3075 (3)	0.39837 (18)	0.34247 (16)	0.0305 (4)	
O5	0.5574 (4)	0.1439 (2)	0.2160 (2)	0.0633 (8)	
O6	0.4884 (5)	−0.0410 (3)	0.2191 (2)	0.0874 (11)	
O1W	0.0433 (3)	0.6507 (2)	0.61730 (16)	0.0332 (5)	
H11	0.105 (4)	0.646 (3)	0.5489 (12)	0.050*	
H12	−0.056 (3)	0.625 (3)	0.626 (2)	0.050*	
O2W	0.2753 (3)	0.6602 (2)	0.41019 (18)	0.0385 (5)	
H21	0.279 (4)	0.593 (2)	0.374 (3)	0.058*	
H22	0.372 (3)	0.650 (3)	0.435 (3)	0.058*	
C1	0.1932 (4)	0.3366 (3)	0.6318 (2)	0.0256 (6)	
C2	0.2383 (4)	0.2132 (2)	0.5588 (2)	0.0230 (5)	
C3	0.1636 (4)	0.1084 (3)	0.6101 (2)	0.0333 (7)	
H3	0.0890	0.1179	0.6854	0.040*	
C4	0.1973 (4)	−0.0096 (3)	0.5522 (3)	0.0389 (7)	
H4	0.1505	−0.0795	0.5892	0.047*	
C5	0.3010 (4)	−0.0224 (3)	0.4391 (3)	0.0350 (7)	
H5	0.3223	−0.1002	0.3981	0.042*	
C6	0.3725 (4)	0.0823 (3)	0.3879 (2)	0.0257 (6)	
C7	0.3471 (3)	0.2024 (2)	0.4442 (2)	0.0210 (5)	
C8	0.4222 (4)	0.3186 (2)	0.3836 (2)	0.0213 (5)	
C9	0.4136 (4)	0.3603 (3)	0.8485 (2)	0.0330 (6)	
H9	0.4684	0.3147	0.7789	0.040*	
C10	0.4522 (4)	0.3043 (3)	0.9478 (3)	0.0379 (7)	
H10	0.5333	0.2229	0.9445	0.045*	
C11	0.3696 (4)	0.3698 (3)	1.0513 (3)	0.0383 (7)	
H11A	0.3934	0.3332	1.1189	0.046*	
C12	0.2507 (4)	0.4911 (3)	1.0534 (2)	0.0340 (7)	
H12A	0.1921	0.5369	1.1225	0.041*	
C13	0.2199 (4)	0.5435 (3)	0.9505 (2)	0.0266 (6)	
C14	0.0971 (4)	0.6732 (3)	0.9424 (2)	0.0281 (6)	
C15	0.0074 (4)	0.7584 (3)	1.0353 (3)	0.0395 (7)	
H15	0.0244	0.7368	1.1080	0.047*	
C16	−0.1067 (5)	0.8752 (3)	1.0185 (3)	0.0474 (8)	
H16	−0.1691	0.9332	1.0799	0.057*	
C17	−0.1276 (5)	0.9055 (3)	0.9093 (3)	0.0483 (8)	
H17	−0.2030	0.9845	0.8961	0.058*	
C18	−0.0351 (5)	0.8171 (3)	0.8207 (3)	0.0396 (7)	
H18	−0.0501	0.8374	0.7474	0.047*	

### Atomic displacement parameters (Å^2^)

**Table:**

	*U*^11^	*U*^22^	*U*^33^	*U*^12^	*U*^13^	*U*^23^
Cu1	0.0282 (2)	0.0265 (2)	0.01756 (19)	−0.00328 (14)	−0.00774 (13)	−0.00078 (12)
N1	0.0379 (14)	0.0317 (14)	0.0318 (13)	−0.0003 (11)	−0.0108 (11)	−0.0096 (11)
N2	0.0292 (12)	0.0285 (12)	0.0210 (11)	−0.0074 (10)	−0.0082 (9)	−0.0014 (9)
N3	0.0317 (13)	0.0270 (12)	0.0242 (11)	−0.0058 (10)	−0.0051 (10)	−0.0004 (9)
O1	0.0316 (10)	0.0304 (10)	0.0225 (9)	−0.0080 (8)	−0.0057 (8)	−0.0061 (8)
O2	0.0400 (12)	0.0493 (13)	0.0274 (11)	−0.0112 (10)	0.0044 (9)	−0.0098 (9)
O3	0.0236 (10)	0.0363 (11)	0.0288 (10)	−0.0104 (8)	−0.0080 (8)	0.0044 (8)
O4	0.0328 (10)	0.0280 (10)	0.0334 (10)	−0.0040 (8)	−0.0167 (9)	0.0093 (8)
O5	0.101 (2)	0.0467 (15)	0.0320 (12)	−0.0231 (15)	0.0086 (13)	−0.0070 (11)
O6	0.125 (3)	0.0627 (18)	0.0617 (18)	−0.0406 (19)	0.0202 (18)	−0.0405 (15)
O1W	0.0281 (11)	0.0455 (12)	0.0278 (10)	−0.0052 (9)	−0.0125 (9)	0.0023 (9)
O2W	0.0421 (12)	0.0405 (12)	0.0355 (12)	−0.0119 (10)	−0.0121 (10)	−0.0007 (9)
C1	0.0316 (15)	0.0300 (14)	0.0164 (12)	−0.0039 (12)	−0.0108 (11)	0.0031 (10)
C2	0.0227 (13)	0.0254 (13)	0.0219 (13)	−0.0026 (11)	−0.0097 (11)	0.0020 (10)
C3	0.0349 (16)	0.0368 (16)	0.0281 (14)	−0.0098 (13)	−0.0073 (12)	0.0056 (12)
C4	0.0441 (18)	0.0285 (16)	0.0461 (18)	−0.0150 (14)	−0.0102 (15)	0.0105 (13)
C5	0.0362 (16)	0.0225 (14)	0.0489 (18)	−0.0047 (12)	−0.0167 (14)	−0.0022 (13)
C6	0.0237 (13)	0.0263 (14)	0.0273 (14)	0.0000 (11)	−0.0107 (11)	−0.0033 (11)
C7	0.0195 (12)	0.0221 (13)	0.0234 (13)	−0.0014 (10)	−0.0116 (10)	0.0015 (10)
C8	0.0264 (14)	0.0238 (13)	0.0145 (11)	−0.0053 (11)	−0.0062 (10)	−0.0023 (10)
C9	0.0373 (16)	0.0332 (16)	0.0279 (14)	−0.0022 (13)	−0.0114 (12)	−0.0013 (12)
C10	0.0413 (17)	0.0339 (16)	0.0432 (17)	−0.0056 (13)	−0.0223 (14)	0.0079 (13)
C11	0.0442 (18)	0.0487 (19)	0.0295 (15)	−0.0182 (15)	−0.0178 (14)	0.0138 (13)
C12	0.0375 (16)	0.0489 (18)	0.0206 (13)	−0.0177 (14)	−0.0094 (12)	0.0010 (12)
C13	0.0285 (14)	0.0326 (15)	0.0217 (13)	−0.0120 (12)	−0.0072 (11)	−0.0002 (11)
C14	0.0280 (14)	0.0323 (15)	0.0252 (14)	−0.0121 (12)	−0.0041 (11)	−0.0018 (11)
C15	0.0438 (18)	0.0445 (18)	0.0281 (15)	−0.0139 (15)	−0.0007 (13)	−0.0085 (13)
C16	0.049 (2)	0.0407 (19)	0.0447 (19)	−0.0076 (16)	0.0027 (16)	−0.0164 (15)
C17	0.048 (2)	0.0313 (17)	0.058 (2)	−0.0008 (15)	−0.0065 (17)	−0.0074 (15)
C18	0.0457 (18)	0.0313 (16)	0.0382 (17)	−0.0026 (14)	−0.0096 (14)	0.0018 (13)

### Geometric parameters (Å, °)

**Table:**

Cu1—O1	1.967 (2)	C3—H3	0.9300
Cu1—O3^i^	2.172 (2)	C4—C5	1.378 (4)
Cu1—O1w	1.994 (2)	C4—H4	0.9300
Cu1—N2	2.029 (2)	C5—C6	1.380 (4)
Cu1—N3	2.013 (2)	C5—H5	0.9300
N1—O5	1.199 (3)	C6—C7	1.403 (4)
N1—O6	1.213 (3)	C7—C8	1.533 (3)
N1—C6	1.480 (4)	C9—C10	1.383 (4)
N2—C13	1.348 (3)	C9—H9	0.9300
N2—C9	1.340 (4)	C10—C11	1.373 (4)
N3—C18	1.339 (4)	C10—H10	0.9300
N3—C14	1.347 (4)	C11—C12	1.382 (4)
O1—C1	1.274 (3)	C11—H11A	0.9300
O2—C1	1.240 (3)	C12—C13	1.390 (4)
O3—C8	1.244 (3)	C12—H12A	0.9300
O3—Cu1^i^	2.1722 (18)	C13—C14	1.483 (4)
O4—C8	1.252 (3)	C14—C15	1.387 (4)
O1W—H11	0.83 (3)	C15—C16	1.374 (5)
O1W—H12	0.83 (3)	C15—H15	0.9300
O2W—H21	0.84 (3)	C16—C17	1.381 (5)
O2W—H22	0.84 (3)	C16—H16	0.9300
C1—C2	1.515 (4)	C17—C18	1.374 (4)
C2—C3	1.393 (4)	C17—H17	0.9300
C2—C7	1.404 (4)	C18—H18	0.9300
C3—C4	1.382 (4)		
			
O1—Cu1—O1W	92.15 (8)	C5—C6—C7	124.0 (3)
O1—Cu1—N3	168.62 (8)	C5—C6—N1	115.6 (2)
O1W—Cu1—N3	88.44 (9)	C7—C6—N1	120.5 (2)
O1—Cu1—N2	95.88 (8)	C2—C7—C6	116.0 (2)
O1W—Cu1—N2	160.36 (9)	C2—C7—C8	121.2 (2)
N3—Cu1—N2	80.22 (9)	C6—C7—C8	122.7 (2)
O1—Cu1—O3^i^	95.27 (8)	O3—C8—O4	128.0 (2)
O1W—Cu1—O3^i^	86.74 (8)	O3—C8—C7	117.1 (2)
N3—Cu1—O3^i^	96.11 (8)	O4—C8—C7	114.9 (2)
N2—Cu1—O3^i^	110.24 (8)	N2—C9—C10	122.1 (3)
O5—N1—O6	121.7 (3)	N2—C9—H9	119.0
O5—N1—C6	120.4 (2)	C10—C9—H9	119.0
O6—N1—C6	117.9 (3)	C11—C10—C9	119.5 (3)
C13—N2—C9	118.6 (2)	C11—C10—H10	120.3
C13—N2—Cu1	114.89 (18)	C9—C10—H10	120.3
C9—N2—Cu1	126.25 (18)	C10—C11—C12	119.0 (3)
C18—N3—C14	118.9 (2)	C10—C11—H11A	120.5
C18—N3—Cu1	125.6 (2)	C12—C11—H11A	120.5
C14—N3—Cu1	115.52 (18)	C11—C12—C13	118.9 (3)
C1—O1—Cu1	108.99 (16)	C11—C12—H12A	120.5
C8—O3—Cu1^i^	134.66 (17)	C13—C12—H12A	120.5
Cu1—O1W—H11	109 (2)	N2—C13—C12	121.9 (3)
Cu1—O1W—H12	116 (2)	N2—C13—C14	114.5 (2)
H11—O1W—H12	112 (2)	C12—C13—C14	123.6 (2)
H21—O2W—H22	109 (2)	N3—C14—C15	121.4 (3)
O2—C1—O1	124.0 (2)	N3—C14—C13	114.6 (2)
O2—C1—C2	118.2 (2)	C15—C14—C13	124.0 (3)
O1—C1—C2	117.7 (2)	C16—C15—C14	119.2 (3)
C3—C2—C7	120.2 (2)	C16—C15—H15	120.4
C3—C2—C1	116.7 (2)	C14—C15—H15	120.4
C7—C2—C1	123.1 (2)	C15—C16—C17	119.2 (3)
C4—C3—C2	121.8 (3)	C15—C16—H16	120.4
C4—C3—H3	119.1	C17—C16—H16	120.4
C2—C3—H3	119.1	C18—C17—C16	118.9 (3)
C3—C4—C5	119.3 (3)	C18—C17—H17	120.5
C3—C4—H4	120.3	C16—C17—H17	120.5
C5—C4—H4	120.3	N3—C18—C17	122.4 (3)
C6—C5—C4	118.8 (3)	N3—C18—H18	118.8
C6—C5—H5	120.6	C17—C18—H18	118.8
C4—C5—H5	120.6		
			
O1—Cu1—N2—C13	−164.61 (18)	C3—C2—C7—C8	176.7 (2)
O1W—Cu1—N2—C13	−51.0 (3)	C1—C2—C7—C8	−2.3 (4)
N3—Cu1—N2—C13	4.59 (18)	C5—C6—C7—C2	−1.3 (4)
O3^i^—Cu1—N2—C13	97.54 (19)	N1—C6—C7—C2	178.2 (2)
O1—Cu1—N2—C9	9.4 (2)	C5—C6—C7—C8	−177.7 (2)
O1W—Cu1—N2—C9	123.1 (3)	N1—C6—C7—C8	1.8 (4)
N3—Cu1—N2—C9	178.6 (2)	Cu1^i^—O3—C8—O4	−14.3 (4)
O3^i^—Cu1—N2—C9	−88.4 (2)	Cu1^i^—O3—C8—C7	166.26 (16)
O1—Cu1—N3—C18	−110.3 (4)	C2—C7—C8—O3	96.7 (3)
O1W—Cu1—N3—C18	−17.1 (2)	C6—C7—C8—O3	−87.1 (3)
N2—Cu1—N3—C18	179.0 (3)	C2—C7—C8—O4	−82.8 (3)
O3^i^—Cu1—N3—C18	69.4 (2)	C6—C7—C8—O4	93.3 (3)
O1—Cu1—N3—C14	68.2 (5)	C13—N2—C9—C10	−0.5 (4)
O1W—Cu1—N3—C14	161.3 (2)	Cu1—N2—C9—C10	−174.3 (2)
N2—Cu1—N3—C14	−2.61 (19)	N2—C9—C10—C11	1.1 (5)
O3^i^—Cu1—N3—C14	−112.15 (19)	C9—C10—C11—C12	−0.5 (5)
O1W—Cu1—O1—C1	−79.31 (17)	C10—C11—C12—C13	−0.7 (4)
N3—Cu1—O1—C1	13.5 (5)	C9—N2—C13—C12	−0.7 (4)
N2—Cu1—O1—C1	82.74 (17)	Cu1—N2—C13—C12	173.8 (2)
O3^i^—Cu1—O1—C1	−166.23 (16)	C9—N2—C13—C14	179.8 (2)
Cu1—O1—C1—O2	−4.2 (3)	Cu1—N2—C13—C14	−5.7 (3)
Cu1—O1—C1—C2	177.93 (17)	C11—C12—C13—N2	1.3 (4)
O2—C1—C2—C3	−26.2 (4)	C11—C12—C13—C14	−179.3 (3)
O1—C1—C2—C3	151.8 (2)	C18—N3—C14—C15	−0.3 (4)
O2—C1—C2—C7	152.9 (2)	Cu1—N3—C14—C15	−178.9 (2)
O1—C1—C2—C7	−29.2 (4)	C18—N3—C14—C13	178.9 (2)
C7—C2—C3—C4	1.7 (4)	Cu1—N3—C14—C13	0.4 (3)
C1—C2—C3—C4	−179.2 (3)	N2—C13—C14—N3	3.5 (3)
C2—C3—C4—C5	−2.8 (5)	C12—C13—C14—N3	−176.0 (3)
C3—C4—C5—C6	1.7 (5)	N2—C13—C14—C15	−177.3 (3)
C4—C5—C6—C7	0.3 (4)	C12—C13—C14—C15	3.3 (4)
C4—C5—C6—N1	−179.2 (3)	N3—C14—C15—C16	0.6 (4)
O5—N1—C6—C5	−175.4 (3)	C13—C14—C15—C16	−178.6 (3)
O6—N1—C6—C5	3.8 (4)	C14—C15—C16—C17	−0.8 (5)
O5—N1—C6—C7	5.1 (4)	C15—C16—C17—C18	0.7 (5)
O6—N1—C6—C7	−175.7 (3)	C14—N3—C18—C17	0.3 (5)
C3—C2—C7—C6	0.3 (4)	Cu1—N3—C18—C17	178.7 (2)
C1—C2—C7—C6	−178.7 (2)	C16—C17—C18—N3	−0.5 (5)

Symmetry codes: (i) −*x*+1, −*y*+1, −*z*+1.

### Hydrogen-bond geometry (Å, °)

**Table:**

*D*—H···*A*	*D*—H	H···*A*	*D*···*A*	*D*—H···*A*
O1w—H11···O2w	0.83 (3)	1.85 (1)	2.660 (3)	166 (3)
O1w—H12···O4^ii^	0.83 (3)	1.90 (1)	2.718 (3)	168 (3)
O2w—H21···O4	0.84 (3)	2.04 (2)	2.830 (3)	158 (3)
O2w—H22···O1^i^	0.84 (3)	2.25 (2)	2.985 (3)	146 (3)
O2w—H22···O3^i^	0.84 (3)	2.35 (3)	2.977 (3)	132 (3)

Symmetry codes: (ii) −*x*, −*y*+1, −*z*+1; (i) −*x*+1, −*y*+1, −*z*+1.
